# Generative AI and the changing meaning of survey responses in digital health research

**DOI:** 10.17179/excli2026-9602

**Published:** 2026-07-29

**Authors:** Pyae Linn Aung

**Affiliations:** 1 Mahidol Vivax Research Unit, Faculty of Tropical Medicine, Mahidol University, Bangkok 10400, Thailand

## ⁯

Online surveys have become fundamental tools in digital health research, public health surveillance, and social and behavioral assessment (Donelle et al., 2023[2]). They are widely used to measure health knowledge, attitudes, risk perceptions, mental health status, treatment adherence, and health-related behaviors, often informing program development and policy decisions. The rapid expansion of smartphone access and internet connectivity has accelerated the use of self-administered online questionnaires across diverse populations. However, the growing availability of generative artificial intelligence (AI) tools introduces a methodological challenge that may alter the interpretation of survey findings (Kuru, 2025[5]).

A fundamental assumption underlying survey research is that responses reflect respondents' own knowledge, beliefs, experiences, or behaviors. Researchers have long recognized sources of measurement error such as recall bias, social desirability bias, and inattentive responding. The emergence of generative AI introduces a different concern. Respondents can now generate coherent, contextually appropriate, and often highly accurate answers using AI systems without necessarily possessing the knowledge or experiences that surveys intend to measure (Afreen et al., 2025[1]; Zhang et al., 2025[9]). Recent evidence suggests that participants may use generative AI tools when responding to survey questions, potentially influencing response content and contributing to greater similarity across responses (Pagallo et al., 2024[6]).

This phenomenon extends beyond deliberate attempts to mislead researchers. AI-powered writing assistance, predictive text, search functions, and conversational interfaces are increasingly integrated into everyday digital environments (Thirunavukarasu et al., 2023[8]). A participant encountering a difficult or unfamiliar question may consult an AI tool to obtain what appears to be the “correct” answer. Even when used with good intentions, such assistance may result in responses that reflect externally generated information rather than the respondent's own understanding or lived experience.

The implications for digital health research may be substantial. First, the validity of survey-based measures may be affected. Knowledge assessments, awareness indicators, or preparedness measures may increasingly reflect access to AI tools and the ability to use them effectively rather than the underlying constructs they were designed to capture. For example, respondents with limited knowledge of disease prevention or treatment may still achieve high scores if they rely on AI-generated answers. Consequently, survey findings may overestimate true levels of population knowledge or awareness.

Second, AI-assisted responding may distort relationships between variables that are central to many behavioral and public health models. Associations between knowledge, attitudes, and practices are frequently used to understand health behaviors and guide interventions. If reported knowledge is increasingly influenced by AI-generated information rather than personal understanding, observed associations may become weaker, stronger, or otherwise different from the true underlying relationships. Such distortions could affect conclusions regarding the determinants of health behaviors and the effectiveness of public health interventions.

Third, AI-assisted responding may complicate the interpretation of population trends and comparisons between groups. Apparent improvements in health literacy, awareness, or preparedness observed over time may partly reflect increased use of AI tools rather than genuine changes in underlying knowledge or behavior. In addition, access to digital technologies, AI tools, language proficiency, and digital literacy varies across age groups, educational levels, occupations, socioeconomic strata, and geographic settings (Dorta-González et al., 2024[3]; Kohnke et al., 2023[4]; Tafazoli, 2024[7]). Consequently, observed differences between groups may increasingly reflect differential access to AI-assisted response generation rather than true differences in the constructs being measured.

These concerns do not suggest that online surveys are no longer useful. Rather, survey methodologies should evolve to accommodate changing digital environments. Several practical approaches may help mitigate this emerging challenge. Researchers may consider using more experiential and context-specific questions that require recall of personal experiences rather than factual information alone. Scenario-based assessments may provide greater insight into applied judgment and decision-making. Consistency checks, response-time monitoring, and authenticity probes may help identify responses that warrant closer scrutiny. Researchers should also consider acknowledging the possibility of AI-assisted responding when interpreting findings from unsupervised online surveys.

Further methodological research is needed to quantify the extent of this issue and evaluate potential mitigation strategies. Comparative studies examining supervised and unsupervised survey administration, different survey formats, and varying levels of AI accessibility could provide valuable evidence regarding the magnitude and direction of potential bias. Ethical guidance may also be needed to address questions of transparency, disclosure, and appropriate use of AI in research participation.

Generative AI is rapidly becoming embedded in everyday digital interactions. As online surveys continue to expand, researchers should recognize that survey responses may increasingly reflect not only what respondents know, believe, or do, but also what digital technologies can help them generate. Anticipating and addressing this challenge will be important for maintaining the validity, interpretability, and usefulness of survey-based evidence in digital health research.

## Declaration

### Artificial Intelligence (AI) - assisted technology

AI-assisted technology was used solely for language editing and improvement of readability. The scientific content, interpretation, and conclusions are entirely the responsibility of the author.

### Conflict of interest

The author declares no conflict of interest.
